# Evaluation of firefighters' heat strain using heart rate during breaks at work

**DOI:** 10.1186/2046-7648-4-S1-A52

**Published:** 2015-09-14

**Authors:** Siyeon Kim, Joo-Young Lee

**Affiliations:** 1 COM:FORT Laboratory, College of Human Ecology, Seoul National University, Seoul, Republic of Korea

## Introduction

Real-time monitoring of firefighters' heat strain during work has been attempted by recording deep body temperature and heart rate (HR). However, monitoring deep body temperature with HR during firefighting is cumbersome and inconvenient for firefighters. Furthermore, monitoring either deep body temperature or HR solely does not reflect full heat strain during firefighting while wearing personal protective clothing with self-contained breathing apparatus (SCBA). Because the capacity of SCBA is limited to 30~45 min, firefighters should take short breaks during firefighting to replace the SCBA. In the US, they provide guidelines to have firefighters take a minimum 10 min break after using a bottle of SCBA, and a minimum 20 min break after using two bottles of SCBA. However, it is more helpful to prevent heat-related illness and diseases of firefighters if we propose a necessary break time based on non-invasively-monitored physiological response. The purpose of the present study was to investigate the possibility of HR as a heat strain index for firefighters during rest periods.

## Methods

Twelve professional male firefighters participated in an experiment wearing firefighters' personal protective equipment (15 kg) with intermittent exercises at an air temperature of 32 °C and 43% relative humidity. Participants began each trial with a 10 min rest and performed two bouts of 15 min exercises on a treadmill at 5.5 km.hr^-1 ^(60% VO_2max_) that were separated by 10 min of seated rest. HR, rectal temperature (T_re_), and oxygen consumption were measured. T_re.break _and ΔT_re.break _were defined as T_re _during rest periods and changes in T_re _during rest, respectively. Linear regression equations were derived between HR and T_re_. Heart rate was expressed as absolute (HR_absolute, _bpm) and relative values (HR_relative, _%HR_max_).

## Results

During rest periods, significant regression equations were derived between HR and T_re; _and between HR and ΔT_re_:

(1) $${\text{T}}_{\text{re}\text{.break}}=0.035\cdot \text{H}{\text{R}}_{\text{relative}}+35.83\left({\text{R}}^{2}=0.722,P<0.05\right)$$

(2) $$\text{Δ}{\text{T}}_{\text{re}\text{.break}}=0.0008\cdot \text{H}{\text{R}}_{\text{relative}}-0.0066\left({\text{R}}^{2}=0.506,P<0.05\right)$$

(3) $${\text{T}}_{\text{r}}\left(t\right)=0.035\cdot \text{\% }HR_{rest}+35.830+\left(8\times \%HR_{rest}+66\right)\cdot 10^{-4}\cdot \text{t}\;\left({\text{R}}^{\text{2}}=0.\text{7}0\text{8},P<0.0\text{5}\right)$$

**Figure 1:**
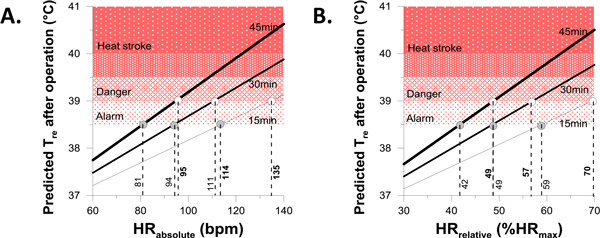
**Reference values of heart rate to commence firefighting operations for 15 min, 30 min, and 45 min**. A: Absolute value of heart rate (bpm), B: Relative value of HR (%HR_max_).

By using equation 3, we calculated HR reference values which can identify when it is safe to continue 15 min, 30 min or 45 min operations after breaks (Figure 1). For example, to continue a 15 min of operation after a break at work without any heat-related illness, HR during the break should be less than 70% HR_max _(39°C T_re _predicted).

## Conclusion

We confirmed the possibility of using a heart rate index during breaks at work to evaluate firefighters' heat strain. Using heart rate during breaks at work, we can determine whether to continue or stop firefighters' operations in hot environments. However, further studies are required to confirm the validity of using a heart rate index in various thermal environments and work intensities of firefighting to determine when it is safe to return to work.

